# Mitigation of perioperative neurocognitive disorders: A holistic approach

**DOI:** 10.3389/fnagi.2022.949148

**Published:** 2022-07-27

**Authors:** Seyed A. Safavynia, Peter A. Goldstein, Lisbeth A. Evered

**Affiliations:** ^1^Department of Anesthesiology, Weill Cornell Medicine, New York, NY, United States; ^2^Department of Medicine, Weill Cornell Medicine, New York, NY, United States; ^3^Feil Family Brain & Mind Research Institute, Weill Cornell Medicine, New York, NY, United States; ^4^Department of Anaesthesia and Acute Pain Medicine, St. Vincent’s Hospital, Melbourne, VIC, Australia; ^5^Department of Critical Care, The University of Melbourne, Melbourne, VIC, Australia

**Keywords:** cognition, anesthesia, perioperative neurocognitive disorders (PND), review, delirium, surgery

## Abstract

William Morton introduced the world to ether anesthesia for use during surgery in the Bullfinch Building of the Massachusetts General Hospital on October 16, 1846. For nearly two centuries, the prevailing wisdom had been that the effects of general anesthetics were rapidly and fully reversible, with no apparent long-term adverse sequelae. Despite occasional concerns of a possible association between surgery and anesthesia with dementia since 1887 (Savage, 1887), our initial belief was robustly punctured following the publication in 1998 of the International Study of Post-Operative Cognitive Dysfunction [ISPOCD 1] study by Moller et al. (1998) in The Lancet, in which they demonstrated in a prospective fashion that there were in fact persistent adverse effects on neurocognitive function up to 3 months following surgery and that these effects were common. Since the publication of that landmark study, significant strides have been made in redefining the terminology describing cognitive dysfunction, identifying those patients most at risk, and establishing the underlying etiology of the condition, particularly with respect to the relative contributions of anesthesia and surgery. In 2018, the International Nomenclature Consensus Working Group proposed new nomenclature to standardize identification of and classify perioperative cognitive changes under the umbrella of perioperative neurocognitive disorders (PND) (Evered et al., 2018a). Since then, the new nomenclature has tried to describe post-surgical cognitive derangements within a unifying framework and has brought to light the need to standardize methodology in clinical studies and motivate such studies with hypotheses of PND pathogenesis. In this narrative review, we highlight the relevant literature regarding recent key developments in PND identification and management throughout the perioperative period. We provide an overview of the new nomenclature and its implications for interpreting risk factors identified by clinical association studies. We then describe current hypotheses for PND development, using data from clinical association studies and neurophysiologic data where appropriate. Finally, we offer broad clinical guidelines for mitigating PND in the perioperative period, highlighting the role of Brain Enhanced Recovery After Surgery (Brain-ERAS) protocols.

## Introduction

Perioperative neurocognitive disorders (PND) are a group of disorders manifest in relation to surgery and anesthesia (Evered et al., 2018a) and encompass former classifications of perioperative cognitive derangements, such as postoperative delirium (POD) and postoperative cognitive dysfunction (POCD). PND are common following anesthesia and surgery, affecting up to 65% of adults over age 65 years (Rudolph and Marcantonio, 2011), and conferring significant morbidity (Suraarunsumrit et al., 2022) and mortality (Roach et al., 1996; Evered et al., 2016; Kok et al., 2017; Relander et al., 2020). Despite the high estimated incidence of PND in older adults, the true incidence is likely higher as most patients do not receive baseline or follow-up neurocognitive testing in the perioperative period and beyond, limiting identification and early intervention. Patients vulnerable to PND can have long-term manifestations: Cognitive decline has been shown to persist for more than 7 years following coronary artery bypass graft surgery (Evered et al., 2016) and can be superimposed on the cognitive decline seen in normal aging (Whitlock et al., 2021). The scope of the problem is only expected to increase as life expectancy increases and more older patients are presenting for surgery than ever before (U.S. Department of Health and Human Services, 2010; McDermott and Liang, 2019).

Although PND are common in older individuals, gaps in knowledge between clinical association studies and neurophysiologic data from humans and animal models prevent a more complete understanding of the mechanisms underlying neurocognitive disorders (NCD). For example, clinical association studies have consistently identified advanced age, low level of education, and pre-existing cognitive impairment as strong risk factors for developing PND (Jones et al., 2006; Monk et al., 2008; Zhang et al., 2019). However, results of meta-analyses offer up to 22 perioperative risk factors for PND (Zhang et al., 2020; Chen et al., 2021), including female gender, operating time, hyponatremia, hypokalemia, anemia, and hypoalbuminemia. Such a multitude of risk factors is unlikely to be clinically useful as associations alone cannot provide a clinical context for disease pathogenesis. More simply, clinical associations can tell us *what* the risk factors are for PND, *but not why* these risk factors contribute to disease pathogenesis. Moreover, the relatively small number of studies and significant degree of heterogeneity in these meta-analyses limit the generalizability of the findings to populations at large. On the other hand, combined neurophysiologic data from animal and human studies have shed light onto mechanisms of NCD pathogenesis and have offered preliminary data for biomarker identification and clinical translation. However, the majority of completed and ongoing clinical trials motivated by these data have not shown clinical efficacy in reducing PND (Safavynia and Goldstein, 2018). Thus, the neurophysiological data tell us *how* PND can manifest, *but not who* is vulnerable with respect to developing PND. Therefore, a holistic approach combining clinical and laboratory data is warranted to understand PND more fully within a generalizable framework, improve recognition, and tailor treatment regimens.

Clinically, the perioperative period is a critical time for identification and assessment of PND, necessitating changes in perioperative workflows and coordinated efforts of multidisciplinary medical professionals. Recently, the American Society of Anesthesiologists has proposed and promoted the concept of a perioperative surgical home (PSH) (Kain et al., 2014; Kwon, 2018), with the aim of supervising all aspects of a patient’s care, from the decision to undergo surgery through recovery. In this model, the role of the anesthesiologist is of paramount importance in ensuring preoperative medical optimization, assessing risk, and coordinating care among a variety of medical professionals, including surgeons, nurses, and medical subspecialists. The PSH model thus provides a platform with which to thoroughly evaluate patients for PND at baseline and at regular follow-up intervals based on risk. Moreover, the PSH model supports inclusion of Brain Enhanced Recovery After Surgery (Brain-ERAS) protocols (Berger et al., 2018; Peden et al., 2021), which may be instrumental in mitigating PND development in the perioperative period.

Here, we review the relevant literature regarding recent developments in PND identification and management throughout the perioperative period. We begin with a brief description of the new nomenclature and highlight changes from previous definitions. We then offer a discussion of PND risk factors, combining data from clinical association studies and neurophysiologic data. We offer broad guidelines for mitigation of PND in the perioperative period using available data. Lastly, we will discuss the role of standardized perioperative practices (i.e., Brain-ERAS protocols) to evaluate and mitigate PND.

## Nomenclature

In 2018, the International Nomenclature Consensus Working Group proposed new nomenclature to standardize identification of and classify perioperative cognitive changes under the umbrella of PND (Evered et al., 2018a). This new nomenclature encompasses the overlapping and heterogeneous definitions of pre-existing cognitive impairment, POD, and POCD. By restructuring criteria for the development of PND with Diagnostic and Statistical Manual-5 (DSM-5) definitions of NCD, the revised nomenclature seeks to align perioperative cognitive changes with NCD in the general population, standardize assessments and identification for PND, and promote cross-specialty communication, all necessary for furthering research and improving clinical care in this area. Specifically, the DSM-5 criteria for NCD include: (1) a modest decline in cognition, defined on at least one cognitive domain, with a subjective deficit (expressed by the patient/family or observed by a clinician) and (2) an evaluation of a patient’s instrumental activities of daily living (IADLs) to classify severity. Additionally, diagnosis of NCD requires that the observed cognitive changes cannot be better explained by a medical condition (e.g., Alzheimer’s disease and cerebral vascular disease) or a psychiatric disorder. Note that these criteria necessitate a neurocognitive battery testing several cognitive domains at repeated intervals, which is likely to uncover previously undiagnosed PND, as the number and timing of neurocognitive tests can influence the identification of PND (Lewis et al., 2006).

**FIGURE 1:**
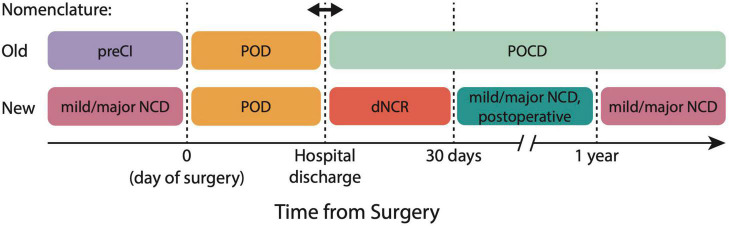
2018 nomenclature for perioperative neurocognitive disorder (PND) classification. The new nomenclature aligns PND definitions with Diagnostic and Statistical Manual-5 (DSM-5) definitions of PND, and reclassifies former conditions of PreCI, POD, and POCD. dNCR, delayed neurocognitive recovery; NCD, neurocognitive disorder; POCD, postoperative cognitive dysfunction; POD, postoperative delirium; PreCI, pre-existing cognitive impairment.

The 2018 nomenclature reclassifies previous diagnoses of POD, POCD, and pre-existing cognitive impairment based on the time scales of development relative to surgery and anesthesia (Figure 1). In the old nomenclature, baseline cognitive impairments on the day of surgery were referred to as pre-existing cognitive impairment (PreCI), acute and fluctuating cognitive impairments during hospital admission were classified as POD, and any residual cognitive impairments following hospital discharge were classified as POCD, even up to 7.5 years postoperatively. In the new nomenclature, (1) any cognitive impairments occurring outside a 1-year window from the time of surgery and anesthesia are classified as mild versus major NCD (unless this is not a *new* diagnosis), (2) criteria and diagnosis of POD remain the same (though can be classified in absence or presence of NCD), and (3) POCD is now classified as delayed neurocognitive recovery (dNCR) if identified within 30 days from surgery, and mild or major NCD, with a postoperative specifier from 30 days to 1 year. The objective criteria for mild NCD (postoperative) are ≥1 standard deviation (SD) to <2 SD below comparative controls or normative data, and for major NCD ≥ 2 SD below controls or norms.

## Risk factors

The combined clinical and neurophysiologic data support risk factors of (1) pre-existing neurologic frailty [see (Evered et al., 2022) for discussion] and (2) perioperative cerebral metabolic stress, which independently and synergistically contribute to PND development. Normal neuronal function is dependent on structurally intact neurons with the available metabolic substrate (i.e., glucose and oxygen) for aerobic cellular respiration. Over time, age-related changes in neuronal metabolism coupled with neurological metabolic insults (e.g., cerebrovascular accidents) and/or neurodegenerative disease contribute to a decline in baseline neurocognitive function (Castelli et al., 2019). During surgery and anesthesia, patients can experience significant cerebral metabolic stress (neuroinflammation, neuroendocrine dysregulation, and oxidative stress), all of which can contribute to functional and/or structural neuronal injury, though it is difficult to tease out the individual effects of surgery versus anesthesia on PND (as clinically, surgery and anesthesia rarely occur in isolation). Taken together, patients who experience perioperative cerebral metabolic stress superimposed on pre-existing neurocognitive frailty are particularly vulnerable to developing PND.

### Pre-existing neurologic frailty: The vulnerable perioperative patient

The most consistently identified preoperative risk factors for developing PND include advanced age, pre-existing neurocognitive impairment, level of education, and alcohol/substance abuse, which are corroborated by meta-analyses (Feinkohl et al., 2017; Zhang et al., 2020; Chen et al., 2021). Aside from these, there is considerable heterogeneity in identified preoperative risk factors due to patient demographics, surgical population, study design, and outcome measures (Marcantonio et al., 1994; Kalisvaart et al., 2006; Monk et al., 2008; Raats et al., 2015; Chaiwat et al., 2019; Kang et al., 2020). Moreover, among these studies, there are no standardized assessments for identifying PND, with heterogeneous definitions for both POD and POCD. While the 2018 nomenclature seeks to resolve this heterogeneity with standardized definitions and criteria, it currently remains unclear whether the identified risk factors are due to individually small effect sizes in heterogeneous populations, which limits the generalizability of identified risk factors across demographic and surgical populations.

Alternatively, commonly identified risk factors for PND have been conceptualized and grouped as markers of pre-existing neurocognitive frailty (Robinson et al., 2012; Culley et al., 2017; Scott et al., 2018; Evered et al., 2020). Here, frailty is defined as the lack of physiological reserve to withstand a physiologic stressor (Clegg et al., 2013), and develops over time from the cumulative effects of cellular and molecular damage in the setting of genetic (e.g., neurodegenerative disease) and environmental factors (e.g., education, physical activity). For example, in normal aging, neuronal glucose availability is altered and glucose uptake is impaired, resulting in overall neuronal hypometabolism (Castelli et al., 2019). Moreover, aging-related alterations in cerebral perfusion and increased blood–brain barrier (BBB) permeability impair toxin removal and reduce mitochondrial metabolic substrate, generating reactive oxygen species. Over time, mitochondria become damaged in response to oxidative stress, rendering neurons even more vulnerable to neurologic insults (Cui et al., 2012; Daulatzai, 2017). The preoperative behavioral manifestations of neurocognitive frailty may be subtle due to neural redundancy and compensation; however, this frailty is likely to be unmasked due to the cerebral metabolic stress of surgery and anesthesia in the setting of low neurocognitive reserve. Indeed, even mildly frail patients (those with limited dependence on others for IADLs) undergoing general surgery have longer hospital stays than age-matched counterparts with increased 30- and 90-day mortality (Hewitt et al., 2015). Note that despite the association between pre-existing frailty and PND, current definitions and assessments of frailty continue to limit its clinical utility. Frailty, by definition, is multifactorial with many interactions between factors. There are a plethora of frailty assessments, with up to 70 different domains assessed in the literature (Rockwood et al., 2005; Hii et al., 2015; Buta et al., 2016), with none considered the “gold standard” assessment (Rockwood et al., 2005), resulting in a wide reported prevalence (10–37%).

### Intraoperative factors: Cerebral metabolic stress

In addition to preoperative neuronal vulnerability, the surgical experience itself confers significant cerebral metabolic stress and confers increased risk for development of PND. Data from human and animal studies provide independent and complementary hypotheses (neuroinflammation, neuroendocrine dysregulation, and oxidative stress) for PND pathogenesis, which are supported by known intraoperative risk factors.

#### Neuroinflammation

The neuroinflammatory hypothesis asserts that (1) surgical trauma causes local inflammation and peripheral pro-inflammatory cytokine cascades, (2) pro-inflammatory cytokines in turn cause BBB disruption and central nervous system (CNS) inflammation, which (3) results in functional and structural neuronal injury. We present a concise overview with preclinical and clinical data where appropriate; for more detailed reviews, please see (Safavynia and Goldstein, 2018; Saxena and Maze, 2018; Saxena et al., 2021).

**FIGURE 2:**
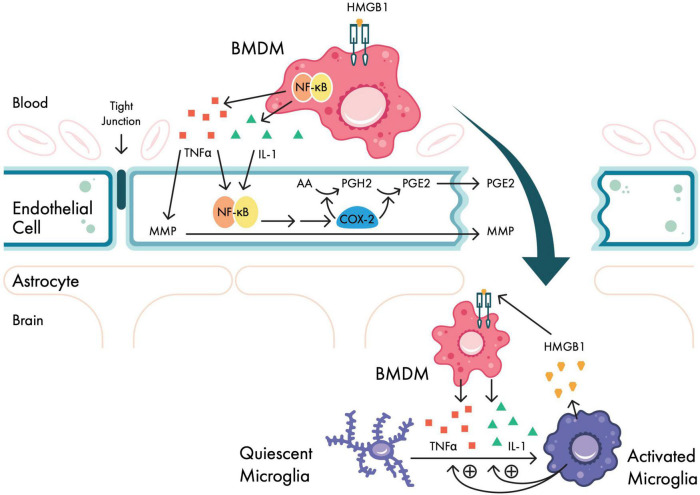
Neuroinflammatory signaling pathways in response to surgical trauma. Bone-marrow derived monocytes (BMDMs) are activated in the periphery in response to surgical trauma via HMGB1. Activated BMDMs upregulate NF-κB expression via second messenger systems, which in turn cause systemic release of pro-inflammatory cytokines IL-1, IL-6, and TNFα. At the blood–brain barrier (BBB), pro-inflammatory cytokines upregulate COX-2 (causing PGE2 production) and MMPs, disrupting BBB permeability. Activated BMDMs then migrate within the CNS, and further cytokine release activates microglia, which in turn activates BMDMs in a reciprocal manner. AA, arachidonic acid; BMDM, bone-marrow-derived monocyte; COX-2, cyclooxygenase 2 isozyme; HMGB1, high mobility group box-1 protein; IL-1, interleukin-1; IL-6, interleukin 6; MMP, matrix metalloproteinase; NF-κB, nuclear factor-kappa B; PGE2, prostaglandin E2; PGH2, prostaglandin H2; TNFα, tumor necrosis factor alpha. Adapted from Safavynia and Goldstein (2018).

In brief, local cellular injury from aseptic surgical trauma causes the passive release of intracellular molecules called damage-associated molecular patterns (DAMPs) (Zhang et al., 2010; Venereau et al., 2015). When exposed to the extracellular environment, DAMPs can activate the immune system by binding to Toll-like receptors (TLRs) on immune cells, including bone-marrow-derived monocytes (BMDMs). In animal models, one such DAMP, high molecular group box 1 protein (HMGB1), has been shown to be upregulated in response to surgery (He et al., 2012); here, extracellular HMGB1 can activate BMDMs (Vacas et al., 2014) either alone or with other DAMPs via TLRs and the receptor for advanced glycation end products (RAGE) (Kierdorf and Fritz, 2013). Through RAGE signaling, BMDMs initiate second messenger cascades that activate nuclear factor kappa B (NF-κB), subsequently causing the systemic release of pro-inflammatory cytokines interleukin-1 beta (IL-1β), interleukin-6 (IL-6), and tumor necrosis factor alpha (TNFα) (Park et al., 2003) (Figure 2). Indeed, several human studies following surgery have shown serum elevations in pro-inflammatory cytokines IL-1β, IL-6, and TNFα (Roth-Isigkeit et al., 1999; Reikeras et al., 2014; Fertleman et al., 2022), as well as C-reactive protein (CRP) (Aono et al., 2007) and erythrocyte sedimentation rate (ESR) (Brdar et al., 2013). Moreover, following surgery, Fertleman et al. (2022) observed concurrent increases of IL-1β, IL-6, and TNFα in cerebrospinal fluid (CSF), suggesting that plasma markers may reflect central inflammatory processes.

In addition to concurrent plasma and CNS cytokine increases following surgery, magnetic resonance imaging (MRI) evidence of BBB disruption has been seen in patients following cardiac surgery (Merino et al., 2013). The increased permeability is believed to be mediated by peripheral, pro-inflammatory cytokines *via* two mechanisms: cyclooxygenase 2 isozyme (COX-2) mediated prostaglandin E_2_ (PGE_2_) synthesis (shown in humans) (Buvanendran et al., 2006) and matrix metalloproteinases (MMPs) (shown in rodents) (Yang et al., 2007), though *in vivo* evidence for the role of MMPs is scant (Rempe et al., 2016). Following surgery, BMDMs (Berger et al., 2019) and pro-inflammatory cytokines are found in human CSF samples after orthopedic (Hirsch et al., 2016) and cardiac (Reinsfelt et al., 2012; Fertleman et al., 2022) surgery, consistent with CNS migration. Within the CNS, activated microglia can amplify neuroinflammation by promoting further BMDM migration into the CNS and BMDM-dependent pro-inflammatory cytokine release (Clausen et al., 2008; D’Mello et al., 2009). However, an observational study in eight male human patients following abdominal surgery showed that despite elevated plasma levels of IL-6, TNFα, and CRP on postoperative days 3–4, there were increases in the anti-inflammatory cytokine IL-10 and decreases in levels of brain translocator protein (TPSO), reflecting a downregulation of microglial activation (Forsberg et al., 2017). Thus, while surgery can initiate peripheral inflammatory responses, the time course of central pro- and anti-inflammatory responses remains unknown. Note that the aforementioned mechanisms are not unique to surgical trauma and have been implicated in multiple human disease states, including sepsis (Manabe and Heneka, 2021), multiple sclerosis (Bucova et al., 2020), Alzheimer’s disease (Reinsfelt et al., 2013; Morgan et al., 2019; Gaikwad et al., 2021), and frontotemporal dementia (Bright et al., 2019).

From a mechanistic perspective, CNS inflammation is postulated to cause cognitive disturbances *via* its actions on hippocampal long-term potentiation (LTP). Normally, hippocampal memory formation is dependent on glutamate signaling through the metabotropic Glu2 (mGlu2) receptor and ionotropic AMPA and NMDA receptors. This is a highly regulated process: During normal, low-frequency stimulation of hippocampal CA1 neurons, NMDA channels are blocked by magnesium (Coan and Collingridge, 1985; Herron et al., 1985). With high-frequency stimulation, NMDA receptors become activated and increase the number and sensitivity of AMPA receptors, which in turn increases synaptic strength and contributes to synaptic plasticity and memory formation (Cooke and Bliss, 2006). The hippocampus has a large number of cytokine receptors, however, and in the presence of pro-inflammatory cytokines IL-1 and TNFα, hippocampal neurons downregulate expression of mGlu2 causing enhanced and dysregulated AMPA/NMDA receptor expression/function and disrupting LTP (Yirmiya and Goshen, 2011). *In vivo*, following tibial surgery in mice, increases in circulating IL-1, IL-6, and TNFα levels are associated with hippocampal inflammation (Cibelli et al., 2010; Terrando et al., 2010; Hu et al., 2018). Circulating HMGB1 can also activate NMDA receptors, causing increased hippocampal glutamate influx and glutamate toxicity. Moreover, TNFα can cause downregulation of γ-aminobutyric acid type A (GABA_A_) receptors (Stellwagen et al., 2005), further shifting the balance toward excitotoxicity, hippocampal neuronal death, and cognitive impairment.

Evidence from human and animal studies further demonstrates that peripheral and CNS inflammation from surgery and anesthesia is associated with cognitive disturbances. In rats, systemic HMGB1 was shown to be sufficient to cause neurocognitive dysfunction (NCD) following partial hepatectomy as demonstrated by increases in the number of errors in the Barnes maze and in levels of anxiety in the open field test (Terrando et al., 2016); conversely, systemic administration of a neutralizing anti-HMGB1 monoclonal antibody prevented the development of NCD (Terrando et al., 2016). Using a tibial fracture surgical model in mice, the pro-inflammatory cytokines IL-1β (Cibelli et al., 2010), IL-6 (Hu et al., 2018), and TNFα (Terrando et al., 2010) have been independently correlated with deficits in associative learning (defined by decreased freezing time in a fear conditioning model). In all three of these studies, inhibiting the cytokine response via monoclonal antibodies, cytokine receptor antibodies, and/or knockout mouse preparations restored cognitive performance. In humans, a meta-analysis of observational studies of inflammatory markers in POD and POCD showed significant increases in plasma IL-6 and CRP concentrations across both states, plasma IL-1 in POCD, and S100 calcium-binding protein β (S100β – a CNS-specific protein reflecting BBB breakdown) in POD (Liu et al., 2018). Similarly, plasma levels of both S100β and neuron-specific enolase (NSE) were elevated in a cohort of 15 patients up to 36 h following coronary artery bypass grafting, and the median increase in NSE was highly correlated (*r* = 0.76; *p* = 0.001) with level of impairment on a neurocognitive battery (Rasmussen et al., 2002). In addition to pro-inflammatory cytokines and markers of BBB disruption, recent small sample (*n* ≤ 30) human observational trials have identified elevated serum and CSF markers of Alzheimer’s pathology following surgery and anesthesia, including amyloid-β(1–42) (Reinsfelt et al., 2013; Danielson et al., 2021), phosphorylated tau (Tang et al., 2011; Evered et al., 2018b; Danielson et al., 2021), and neurofilament light chain (NfL) (Forsberg et al., 2017; Evered et al., 2018b; Danielson et al., 2021). However, none of these studies were able to determine an association between these biomarkers and neurocognitive endpoints. In a larger cohort of patients (*n* = 59) receiving combined spinal and general anesthesia for elective total hip replacement, Evered et al. (2016) found a significantly lower CSF amyloid-β(1–42) level in patients with POCD (as tested by neurocognitive battery) at 3 months following surgery, noting the potential for a CSF biomarker of NCD but again calling into question the time course of central pro- and anti-inflammatory responses (cf. Forsberg et al., 2017). In a similarly sized cohort (*n* = 54) of patients undergoing major elective surgery, postoperative delirium was associated with higher baseline serum NfL, and higher serum NfL at postoperative day 2 was associated with more severe delirium [as measured by the Confusion Assessment Method (CAM) and CAM-S (severity)] (Fong et al., 2020). Moreover, while serum NfL was elevated in all patients following surgery, the patients with delirium had significantly higher serum NfL levels at 1 month following surgery (Fong et al., 2020).

Clinically, a recent meta-analysis of randomized controlled trials that examined perioperative inflammation after surgery and general anesthesia showed an increase of pro-inflammatory biomarkers postoperatively (O’Bryan et al., 2022). The meta-analysis included 23 studies (one of which was pediatric patients) with 1,611 participants and investigated mean inflammatory biomarker levels IL-6, IL-10, TNFα, and C-reactive protein (CRP) following surgery. Specifically, mean levels of TNFα and IL-6 were increased immediately following surgery; TNFα levels returned to near baseline levels at 12–24 h following surgery, and IL-6 levels were elevated until 48–96 h following surgery. IL-10 increased slightly for 12–24 h following surgery, and CRP levels increased from 12–96 h following surgery. Despite the noted increases in pro-inflammatory cytokines associated with surgery and anesthesia, the correlation between inflammatory biomarkers and development of PND was only assessed in five studies and was inconsistent. Among these five studies, a meta-analysis was unable to be performed due to heterogenous cognitive outcome reporting. Notably, the observed biomarker increases were independent of anesthetic choice (propofol vs. sevoflurane) at all time points measured, suggesting that anesthesia itself may not be the primary driver of neuroinflammation (cf. see Section “Interaction effects – The role of surgery and anesthesia”).

#### Neuroendocrine dysregulation

In addition to neuroinflammation, the hypothalamus responds to surgical stress by releasing corticotrophin-releasing hormone (CRH), which acts on the pituitary gland to release adrenocorticotrophic hormone (ACTH) and subsequent glucocorticoid (GC) release from the adrenal cortex (Maldonado, 2013; Manou-Stathopoulou et al., 2019). Elevations in circulating GC levels can prevent neuronal glucose uptake, rendering neurons metabolically vulnerable and prone to oxidative stress (Sapolsky and Pulsinelli, 1985); moreover, GCs have been shown to enhance ischemic and seizure-induced neuronal injury [(Tombaugh et al., 1992); and see Maldonado (2013) for a comprehensive review]. The hippocampus has one of the highest concentrations of GC receptors among brain regions (Sapolsky et al., 1984), providing a rationale for acute memory disturbances following surgical stress (i.e., delirium) and a subsequent predilection for long-term cognitive decline. This may explain why hyperglycemia and type 2 diabetes have been identified as risk factors for PND (Hermanides et al., 2018); both prolonged exposure to circulating GCs and impaired glucose uptake can precipitate cognitive decline (Goosens and Sapolsky, 2007). When superimposed on the prolonged exposure to GCs and impaired glucose uptake in normal aging (Castelli et al., 2019), profound neuronal hypometabolism and vulnerability to oxidative stress can ensue.

#### Oxidative stress

A third mechanism by which surgery can cause cognitive derangements in the perioperative period and beyond relates to oxidative stress and antioxidant depletion, which causes functional silencing of neurons and cognitive alterations. Here, the high metabolic demands associated with surgical trauma outstrip metabolic reserve in myriad ways, including sympathetic activation from surgical stress, hypoxia, and ischemia-reperfusion injury in the perioperative period (Maldonado, 2013); regardless of etiology, these factors collectively contribute to reduced cerebral oxidative metabolism and reactive oxygen species (ROS) generation. The most vulnerable brain regions are those with high metabolic activity, including the hippocampus and the posteromedial complex, a cortical region involved in resting-state networks that are critical for attention and awareness. From this logic, it is not surprising that clinical association studies identify hypoglycemia, intraoperative anemia, and electrolyte disturbances as risk factors for PND development (Zhang et al., 2020; Chen et al., 2021). Further, the generation of ROS can directly contribute to neuronal damage and disrupt BBB integrity (Abdul-Muneer et al., 2015), converging on the pathophysiology of neuroinflammation and neuroendocrine dysregulation. A recent observational arm of a clinical trial (Billings et al., 2016) highlights the convergence of neuroinflammation and oxidative stress on developing POD; in this study, the authors measured biomarkers of oxidative stress (isoprostanes and isofurans) and BBB disruption (S100β) in patients following cardiac surgery (Lopez et al., 2020). The authors showed that increased serum isoprostane and isofuran levels were associated with POD, and this association was stronger in patients with elevated S100β levels.

The functional neuronal consequences of oxidative stress are manifest in a burst-suppression pattern on electroencephalography (EEG), supported by human data and biophysical mathematical models. Burst-suppression (BS) is an EEG pattern reflecting alternating periods of neural activity (bursts) followed by low-voltage quiescence (suppression); BS is seen in a number of conditions with reduced cerebral metabolic rate of oxygen consumption (CMRO_2_), including hypothermia (Westover et al., 2015), hypoxemia (Liu et al., 2006), post-cardiac arrest (Forgacs et al., 2020), and deep levels of GABA_A_-receptor dependent anesthesia (Shanker et al., 2021), which are likely mediated through direct inhibition of ATP synthesis and blocking of the mitochondrial electron transport chain (Berndt et al., 2018; Kishikawa et al., 2018). A recent mathematical model using a network of Hodgkin-Huxley neurons and described by equations for ATP-gated potassium channel (K_ATP_) current and sodium/ATP exchange showed that BS arises when ATP generation is decreased by 50%, with further decreases in ATP availability extending the duration of BS (Ching et al., 2012). In this model, the decrease in intracellular ATP causes an increase in K_ATP_ channel conductance and K^+^ efflux, hyperpolarizing neurons and preventing spiking activity. As ATP is regenerated, K_ATP_ channels close, increasing the resting membrane potential, and neuronal bursting activity resumes.

Despite conflicting data, there is evidence linking BS to poor cognitive outcomes in certain patient populations, consistent within an oxidative stress framework. For example, the presence of BS during anesthetic maintenance has been shown to be associated with an increased risk of POD (Fritz et al., 2016; Hesse et al., 2019), with longer BS durations seen in delirious versus non-delirious patients (Evered et al., 2021). However, the ENGAGES trial which compared EEG-guided anesthesia versus usual anesthetic care in 1,232 patients at a single hospital showed no change in POD rate when limiting intraoperative BS duration (Wildes et al., 2019). It is important to note that there was BS in both arms of this study (7 min in EEG group compared to 13 min in within the usual care group), raising the possibilities that either a 6-min difference is too small an effect size, or that any degree of BS is pathologic, though the latter is unlikely as in the Hesse study (2019), induction BS was not found to have an association with POD. Taken together, it is possible that the downstream cognitive effects may only occur after a threshold level of burst suppression. It is known that advanced age is associated with increased propensity for BS at lower sedation levels (Purdon et al., 2015), which may reflect the combined effects of oxidative stress on pre-existing neurologic frailty. This may also explain why Shortal et al. (2019) found no correlation between BS duration and cognitive deficits in a series of young healthy human volunteers aged 22–39.5 years. In addition to possible threshold effects, these volunteers may be less susceptible to the effects of BS, as they presumably have less baseline neurocognitive frailty and more cerebral metabolic reserve.

In addition to population heterogeneity, the conflicting data seen in clinical studies may arise from (1) the use of processed EEG (pEEG) monitors to identify BS, (2) heterogeneities among BS detection performance in pEEG monitors, and (3) the use of proprietary indices available on pEEG monitors as a proxy for burst suppression. Classically, pEEG monitors (BIS™, SedLine^®^) display raw EEG data as well as time- and frequency-domain parameters including burst suppression ratio, spectral edge frequency, and artifact detection. Using proprietary algorithms, pEEG monitors report indices with recommended ranges: [i.e., BIS™ Bispectral (BIS) index, SedLine^®^ Patient Safety Index (PSI), Entropy™ state and response entropy] to encapsulate data and provide clinicians with a single number to estimate anesthetic depth (Eagleman et al., 2021). Almost all clinical studies on BS have used the BS calculations provided by the pEEG monitor and not raw EEG data, however, it is known for example that BS as reported by the SedLine^®^ monitor underestimates BS compared to visual analysis (Muhlhofer et al., 2017). Moreover, in a head-to-head comparison of BIS™, SedLine^®^, and Entropy™ modules using identical EEG data, there is considerable variability in the EEG suppression detection across devices (Eagleman et al., 2021), limiting the interpretation of clinical trials and the generalizability to clinical practice. Finally, BS is often inferred in clinical studies using proxy indices such as BIS and PSI, with group comparisons often defined with respect to these indices and not BS itself (Evered and Goldstein, 2021). Though depth of anesthesia indices are shown to correlate with BS at low values (Bruhn et al., 2000), the indices lag real-time data (Kreuzer et al., 2012), are influenced by pharmacologic agents such as neuromuscular blockade (Schuller et al., 2015), and have been shown to be discordant with adequate and stable end-tidal sevoflurane concentrations during maintenance (Epstein et al., 2018), further limiting the utility of the data. Thus, BS and pEEG indices are not interchangeable, nor do the indices themselves consistently reflect the patient’s brain state accurately, and any interpretation of clinical data should be made in the context of these limitations.

### Interaction effects – The role of surgery and anesthesia

Important work begins to disentangle the relationship between biomarkers for inflammation and neuronal injury and the intertwining of anesthetic exposure and surgery (Deiner et al., 2020). Here, the impact of general anesthetic administration (propofol 2 mg/kg i.v. for induction; sevoflurane 1.4–1.8% for 5 h maintenance) in essentially healthy human volunteers (American Society of Anesthesiologists Physical Status 1 or 2) on serum biomarkers in the *absence* of surgery were measured. Following 5 h of sevoflurane administration at age-adjusted depth of 1 minimum alveolar concentration (MAC), tau, neurofilament light (NF-L), and glial fibrillary acidic protein (GFAP) were significantly decreased relative to baseline while plasma TNF-α and CRP were unchanged. Superficially similar to O’Bryan et al. (2022), plasma IL-6 was significantly increased following sevoflurane exposure, but not by a biologically relevant amount (<1 pg/ml). The study by Deiner et al. (2020) does have limitations, however (Evered and Scott, 2020), raising in theory that the negative results reported may represent a Type II error (i.e., failure to reject a false null hypothesis); that said, it at least presents the possibility that anesthetic exposure in and of itself in healthy adult humans *may not* produce a pro-inflammatory state.

Behaviorally, there is recent evidence that anesthesia in the *absence* of surgical intervention does not produce neurocognitive impairment. In a recent single-center cohort study, healthy adult volunteers 40–80 years old (*n* = 71, mean age 58.5 years, 56% male, 44% female) with no underlying cognitive dysfunction underwent cognitive testing before and at multiple time points after 2 h of general anesthesia (propofol weight and age-adjusted dose for induction followed by sevoflurane age-adjusted depth of 1 MAC for maintenance titrated to BIS level of 40–60) (Baxter et al., 2022). As described, “the primary outcome was recovery to baseline on the Postoperative Quality of Recovery Scale (PQRS) cognitive subtest; …secondary outcomes were measures from in-depth neuropsychological testing that covered the domains of executive function and attention, episodic memory, language, processing speed, and working memory; instruments for this testing were the National Institutes of Health (NIH) Toolbox Cognitive Battery and paper-and-pencil neuropsychological tests from the Alzheimer’s Disease Research Center Uniform Dataset Battery: Trail Making Test (parts A and B), Logical Memory (immediate and delayed recall), and Category Fluency as well as the California Verbal Learning Test (CVLT).” The authors found no association on any measure between age (by decade) and time to cognitive recovery within 30 days from anesthetic exposure. These results are congruent with the hypothesis that neuroinflammation secondary to surgical-induced peripheral inflammation is the key etiologic factor contributing to adverse neurocognitive function in the postoperative period. These results should be reassuring to healthy older patients presenting for surgery that anesthesia *per se* will not result in neurocognitive deficits.

In agreement with the proposition that surgery, and not anesthetic exposure, is the prime etiologic force contributing to PND are the results from two recent studies in which different anesthetic regimens were utilized in the setting of intraabdominal laparoscopic surgery (Li Y. et al., 2021) and surgical repair of fragility hip fractures (Li T. et al., 2021). In patients ≥60 years of age undergoing elective laparoscopic abdominal surgery, the rate of neurocognitive impairment [measured 5–7 days after surgery using the neuropsychological test battery from the original International Study of Post-Operative Cognitive Dysfunction paper – (Moller et al., 1998)] was the same between subjects in the inhalational (sevoflurane 1 – 1.5 MAC + remifentanil 0.1 – 0.5 μg/kg/min) anesthesia group versus those in the intravenous (propofol 50 – 150 μg/kg/min + remifentanil 0.1 – 0.5 μg/kg/min) anesthesia group (*per* protocol analysis; 20.8% vs. 16.8%, respectively, *P* = 0.279; intention-to-treat analysis; 21.4% vs. 17.2%, respectively, *P* = 0.245) (Li Y. et al., 2021). Among the many biomarkers measured, only IL-6 was found to correlate positively with the risk of neurocognitive impairment; the other biomarkers did not correlate in either direction. These data again suggest that the choice of anesthetic is not an independent risk factor for the development of impaired neurocognitive function following surgery (at least in the short-term). The results of the RAGA (Regional Anesthesia vs. General Anesthesia) study – a multicenter, pragmatic, randomized allocation-concealed, open-label, parallel-group, multicenter trial at 9 university teaching hospitals in southeastern China (Li T. et al., 2021) – also support that hypothesis. Here, inclusion criteria were age ≥65 years and surgical repair of a femoral neck, femoral head, intertrochanteric, or subtrochanteric fracture. Randomization was to one of two anesthetic regimens: neuraxial blockade (spinal, epidural, or combined spinal epidural) *without any supplemental sedation* or general anesthesia using intravenous induction agents and maintained using intravenous or inhalational anesthetic agents per practitioner preference. The incidence of delirium during the first 7 days following surgery was the primary outcome measure and was measured using the Confusion Assessment Method (CAM), along with multiple secondary outcome measures (episodes, severity, and subtypes of delirium; the worst pain score over the first postoperative 7 days; length of hospitalization; 30-day all-cause mortality; predefined criteria for adverse events; 6-month and 1-year follow-up for delirium incidence, type, and severity; cognitive function, quality of life, and mortality; and hospitalization cost). The incidence of delirium in the immediate postoperative period was comparable between the two groups [Regional: 29/471 (6.2%), General: 24/471 (5/5%); Unadjusted Risk difference (95% confidence interval) – 1.2 (−1.7 to 3.8), *P* = 0.48]. There were no statistical differences between the two groups for any of the secondary outcomes. It is worth noting that the difference in the incidence of neurocognitive dysfunction between the two Li T. et al. (2021) and Li Y. et al. (2021) studies reflects the fact that they were measuring fundamentally different aspects of neurocognitive function, global cognitive function vs. delirium. That said, the fact that the incidence of delirium in the neuraxial and general anesthesia groups in an at-risk population (i.e., those ≥65 years of age) was comparable, suggests that delirium is a consequence of surgical intervention, *not exposure to general anesthetics*. This interpretation also accords with the results of Baxter et al. (2022) who found no association on any measure of cognitive function between age (by decade) and time to cognitive recovery within 30 days from anesthetic exposure.

## Management

We propose a perioperative management scheme to mitigate PND based on the aforementioned hypotheses, paying particular attention to the modifiable preoperative and intraoperative risk factors. Preoperatively we advocate for baseline neurocognitive assessments, especially in high-risk patients, with management centered on optimizing medical comorbidities to preserve cognitive function. Intraoperatively, we posit that cerebral oximetry and EEG are key biomarkers for identifying oxidative stress and neuronal dysfunction, and can be used to tailor anesthetic regimens to preserve and optimize neural function.

### Preoperative cognitive optimization

Preoperative identification of high-risk patients should incorporate patient factors, surgical factors, and pharmacologic factors, ideally well in advance of surgery so that modifiable risk factors can be optimized (Atkins et al., 2021; O’Gara et al., 2021; Peden et al., 2021). In addition to age and pre-existing medical comorbidities, assessing for patient risk factors should include a review of a patient’s post-anesthesia history (if available), as well as a sleep history (Wang et al., 2021), with particular attention to sleep disturbances from pain and/or depressive symptoms, both shown to increase POD risk (O’Gara et al., 2021). It is important to consider the surgery being performed, with attention to surgical pain and/or opioid requirements, anticipated blood loss, and the potential for a prolonged postoperative course. Non-opioid, multimodal pain control should be utilized as much as possible. Pharmacologic assessment is helpful in identifying polypharmacy, which may independently contribute to neurocognitive decline, particularly POD (Duprey et al., 2022; Zietlow et al., 2022). Baseline cognitive screening should be routine in order to identify high-risk patients, who should then undergo full baseline neurocognitive assessment to track PND over multiple neurocognitive domains and initiate early intervention.

As we move toward a perioperative surgical home model, the preoperative period will become even more instrumental in optimizing patients to mitigate PND. Beginning with cognitive screening and baseline neurocognitive assessments, the anesthesiologist should discuss the likelihood of developing PND with the patient *and family members*: by engaging families, we can increase awareness and expectation, and provide a support system for postoperative cognitive therapy if needed. Additionally, family members can be active helpers implementing the Hospital Elder Life Program (HELP) strategies known to reduce the incidence of delirium by up to 40% (Hshieh et al., 2018). There is some evidence that prehabilitation and rehabilitation may reduce the incidence of PND, particularly POD, although evidence is scant. Cognitive training has been shown to have a positive effect on PND (Humeidan et al., 2021) and multi-component pre and postoperative studies are underway (Atkins et al., 2021). Depending on the patient’s medical history, it may be necessary to involve other medical team members, including geriatricians, neurologists, and physical therapists to optimize a patient’s preoperative medical health and functional status. A tailored anesthetic plan should be made, focusing on cognitive protective strategies; this may include avoiding neuromodulatory medications including benzodiazepines and anticholinergics, or the use of multimodal pain management strategies to reduce the total opioid dose.

### Intraoperative interventions

During the intraoperative period, attention should be placed on mitigating the precipitants of cerebral metabolic stress and providing conditions necessary for maintaining neuronal structure and function. While biomarkers can be tracked reflecting neuroinflammatory (S100β and pro-inflammatory cytokines IL-1, IL-6, TNFα), neuroendocrine dysregulation [cerebral glucose uptake *via* fluorodeoxyglucose (FDG) imaging], and oxidative stress (cerebral oximetry, EEG) hypotheses for PND development, of these cerebral oximetry and EEG are readily available, dynamically observable, and clinically actionable in the intraoperative period.

The primary goal of intraoperative management in mitigating PND is to preserve neuronal structure and function in the setting of multiple metabolic stressors. Thus, focus should be placed on ensuring adequate metabolic substrate (i.e., glucose and oxygen) for neurons. Hypoglycemia is rare in the intraoperative period but should be assessed in patients at increased risk for developing PND; more broadly, an adequate oxygen supply is critical for oxidative phosphorylation and ATP production. To this end, it is prudent to maintain organ perfusion by avoiding hypotension and hemodynamic variability. This may be achieved by proper positioning, lower doses of anesthetic, and pharmacologic hemodynamic support as needed. As PND is related to global and local cerebral perfusion, frontal cerebral oximetry may be useful in assessing frontal cortical oxygenation (Holmgaard et al., 2019; Uysal et al., 2020). It is important to note that while cerebral oximetry can provide absolute values of oxygen saturation (Matcher and Cooper, 1994), the correlation between local oxygen saturation and neuronal function is less well understood; thus cerebral oximetry is more effective as a relative saturation monitor. Baseline measurements should be noted and interventions can be adapted to changes in cerebral oxygen saturation.

By contrast, EEG provides a more direct and high-frequency measurement of functional neuronal population activity. Though EEG features of NCD are a rapidly expanding area of research, for now, hypothesis-driven clinical data advocate for (1) limiting intraoperative burst suppression and (2) promoting frontal alpha EEG power, particularly on emergence (but see caveat below). As noted above, BS is closely linked to oxidative stress (Ching et al., 2012), and BS susceptibility is associated with increasing age (Purdon et al., 2015). Thus, it is reasonable to monitor patients for burst suppression via EEG or pEEG devices. As described in Section “Oxidative stress,” for many pEEG devices (BIS™, SedLine^®^), the presence of BS results in a pEEG index lower than the recommended range for general anesthesia (Bruhn et al., 2000; Bruhn, 2002), though BS itself is relatively simple to recognize and has been shown to be feasible for anesthesia providers with limited training (Bottros et al., 2011; Bombardieri et al., 2020). Thus, we recommend titrating anesthetic agent to reduce the amount of burst suppression (*via* calculated BS metrics and/or visual identification) as much as possible, especially in neurocognitively frail patients. More recently, the relative risk of developing POD has been shown to be influenced by the EEG content on emergence (Hesse et al., 2019); specifically, EEG with high alpha (α: 8–12 Hz) power on emergence (“spindle dominant slow-wave anesthesia”) has been associated with the lowest relative risk for POD. This is postulated to be due to thalamocortical interactions (Vijayan et al., 2013), though its neurophysiological significance in relation to NCD remains unknown. Though there is limited published evidence on how to promote alpha power on EEG, reducing anesthetic depth (Kreuzer et al., 2017) and providing adequate analgesia (Garcia et al., 2021) have shown utility and have motivated an ongoing clinical trial to assess the intraoperative modulation of alpha power and its relation to POD (clinicaltrials.gov identifier NCT04443517).

### Role of enhanced recovery after surgery protocols

**FIGURE 3:**
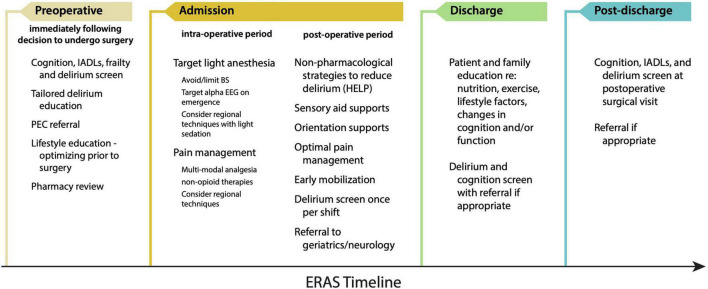
Proposed Brain-ERAS pathway for the prevention and mitigation of PND. HELP, Hospital Elder Life Program; IADL, instrumental activity of daily living; PEC, pre-anesthesia evaluation clinic.

Most medical institutions across the United States have implemented Enhanced Recovery After Surgery (ERAS) protocols for a variety of surgical procedures (Ljungqvist et al., 2021; Gillis et al., 2022). In brief, these protocols target early mobilization, multi-modal, non-opioid pain control, and early discharge from hospital. From a brain health perspective, brain ERAS should not be a stand-alone ERAS protocol, but rather a ‘Brain-ERAS’ protocol that is implemented *within* other ERAS protocols. Such a protocol could include recommendations based on hypotheses of PND pathogenesis: For example, dexamethasone is a commonly used agent for postoperative nausea and vomiting (PONV) prophylaxis (De Oliveira et al., 2013) or prevention of post-extubation airway obstruction (Lee et al., 2007). While useful, dexamethasone administration may not be necessary for many patients, and may actually precipitate harm in a neurocognitively frail patient, especially when other antiemetic agents are available. Moreover, gabapentinoids (pregabalin and gabapentin) are argued to decrease postoperative opioid requirements and attenuate the risk of developing persistent postsurgical pain (PSP), but recent meta-analyses indicate that the acute opioid sparing effects of gabapentinoids are at best only modest (Verret et al., 2020) and the diminution of PSP absent (Carley et al., 2021). Conversely, the centrally-acting sedative effects of gabapentinoids have drawn into question their utility overall (Kharasch et al., 2020), and are likely to confer an increased risk of PND in a neurocognitively frail population. Figure 3 demonstrates an overview of a Brain-ERAS protocol for each stage of the patient journey, commencing pre-operatively with the decision to undergo surgery through to post-discharge.

#### Opioid-sparing strategies

It has been suggested that non-narcotic analgesics may be the preferred approach to perioperative analgesia in the geriatric patient population (Wilson et al., 2022) as opioid administration has been associated with increased rates of POCD (Awada et al., 2019) and possibly POD (Clegg and Young, 2011). Conversely, pain, especially severe pain, increases the risk for delirium [for review, see O’Gara et al. (2021)], and there is clearly a risk-benefit assessment that must be considered as opioid analgesics are highly effective in relieving acute pain.

Given the necessity and utility of opioid analgesics, they are unlikely to escape clinical practice in the near future, and within this context, it has been hypothesized that if opioids are to be used, shorter-acting opioids may be less likely to induce POD. Remifentanil is a potent, ultra-short acting synthetic μ-opiate receptor agonist with no meaningful active metabolites (Westmoreland et al., 1993; Michelsen and Hug, 1996; Egan et al., 2004); sufentanil has many similar properties with several notable exceptions, among which is that has a significantly longer plasma half-life [∼20 min vs. <5 min; (Ogura and Egan, 2019)]. In one study of subjects undergoing supratentorial craniotomy for tumor resection, individuals randomized to receive remifentanil demonstrated a lower incidence of early cognitive impairment (as measured on the Short Orientation Memory Concentration Test [SOCMCT; a validated measure of cognitive impairment; (Katzman et al., 1983)] and the Rancho Los Amigos Scale [RLAS; a tool designed to measure and track cognitive recovery following traumatic brain injury; (Hagen et al., 1980)] than did subjects randomized to receive sufentanil (Bilotta et al., 2007). In contrast, Martorano et al. (2008) reported that in a comparable subject population, subjects randomized to receive sufentanil (as compared to remifentanil) had significantly better SOMCT scores at 15 and 180 min postoperatively (*P* < 0.05) whereas no difference between groups was detected on the RLAS. The argument in favor of remifentanil as the preferred opioid for the prevention of POD is further challenged by the observation that in subjects with ischemic heart disease undergoing elective coronary artery bypass graft surgery, there was no difference in postoperative neurocognitive function [measured on days 1, 4, and 30 following surgery using the Palo Alto Veterans Affairs (VA) Cognitive Questionnaire (PAVA-CQ)] [a battery of 30 simple questions to assess memory and concentration; (Ashford et al., 2022) in subjects randomized to receive either remifentanil or sufentanil (Rasmussen et al., 2016)].

The heterogeneity apparent in these studies (most notably surgical intervention, test selection, and timing of testing) highlights the difficulty in discerning cause and effect. Not surprisingly, a systematic review on the risk of delirium in older subjects (age >60 years) as a function of opioid selection concluded that “there are no convincing data that the risk of delirium in elderly patients is different for the various types of opioids” (Swart et al., 2017). Critically, a secondary analysis of prospectively collected data from the Successful Aging after Surgery (SAGES) study [original inclusion criteria: age ≥70 years old, English-speaking, able to communicate verbally, scheduled to undergo elective surgery (total hip or knee replacement; lumbar, cervical, or sacral laminectomy; lower extremity arterial bypass surgery; open abdominal aortic aneurysm repair; or colectomy) at 1 of 2 Harvard-affiliated academic medical centers with an anticipated length of stay ≥3 days, and availability for in-person follow-up interviews; (Schmitt et al., 2012)] failed to detect any evidence of an association between POD and postoperative opioid administration (adjusted hazard ratio [aHR], 0.82; 95% CI, 0.62–1.11); the possible exception to this appears to be meperidine (Fong et al., 2006; Duprey et al., 2022). The lack of an overall association between narcotic administration and POD was unlikely to be a type II error as they did observe that postoperative hospital benzodiazepine use was associated with greater delirium (aHR, 3.23; 95% CI, 2.10–4.99) (Duprey et al., 2022). While there may be entirely valid reasons for limiting opioid administration postoperatively over concerns for diversion and misuse/abuse of opiate pain relievers (Alam et al., 2012; Brummett et al., 2017; Hah et al., 2017; Han et al., 2017), these data would suggest that the decision to administer (or not administer) perioperative opioids should not be based on the goal of minimizing the risk of POD.

#### Multi-component interventions/prehabilitation

There have been a number of studies investigating multi-component interventions to reduce PND. Some are solely based on the preoperative period and comprise strategies for “prehabilitation” or optimization of the patient in preparation for surgery. Some span the entire perioperative period (Humeidan et al., 2021). Despite the name, most only assess one type of intervention, such as cognitive training or physical exercise. Research in the general community investigating Alzheimer’s disease and related dementias has demonstrated some effectiveness of these interventions in improving cognitive function, or at least reducing decline, however, compliance and assessment are difficult. It is also likely there is a synergistic effect of interventions, suggesting a superior effect with multiple interventions. While such interventions are currently advocated for surgical patients (Hughes et al., 2020), with the exception of the well-validated HELP interventions (Hshieh et al., 2018) there remains scant evidence of the effect of multi-component interventions to reduce PND. The ProTECT Trial is an Australian study currently recruiting patients to be randomized to routine clinical care versus a multi-component intervention to reduce PND. The intervention is complex and is described in further detail in the published protocol (Atkins et al., 2021).

## Future directions

Just as the EEG is important in the intraoperative management of at-risk patients, the role of other biomarkers is going to be very important in understanding the complex pathophysiology of PND. Inflammatory biomarkers (*vide supra*) are gaining increasing traction as potential factors associated with PND, which may be useful to assist in identifying at-risk individuals, as well as understanding disease progression, at least in the short-term. Other potentially valuable plasma biomarkers include neurofilament light (NfL) and tau, as well as several tau variants. NfL has been shown to increase significantly during anesthesia and surgery (cf. see Section “Neuroinflammation”), to levels similar to those observed with acute-traumatic brain injury (aTBI) in sports players (Evered et al., 2018b). In addition, increasing levels of NfL (Casey et al., 2020) and tau (Ballweg et al., 2021) during the perioperative period have been demonstrated to be associated with an increased risk of POD. Ultimately, combining the data offered from plasma biomarkers with a more sensitive assessment and understanding of pEEG waveforms are currently an area of significant investigation which may assist in unraveling the complex mechanisms underlying PND, leading to preventive strategies and interventions.

## Conclusion

The last two centuries have seen extraordinary progress in both surgery and anesthesiology; as a team, we provide meaningful care to a patient population that is older with more extensive comorbidities than ever before. In years past, the hallmark of a “successful” general anesthetic was having the patient extubated at case end and transferring the patient alert, comfortable, and hemodynamically stable to the next stage in their recovery. While those short-term goals are still laudable, it is clear that they are not sufficient; there is an increasing appreciation that what is at least as important, if not more so, are their intermediate- to long-term effects on a patient’s health and well-being. These longer-term outcomes must necessarily include preservation of neurocognitive function. When deficits in such function have been detected, the default response has been to blame the drugs as it is self-evident that general anesthetics disrupt normal cognitive processes to produce the desired amnestic and unconsciousness states that are their hallmarks. As recent data suggest, the causal mechanisms underlying short-term, and possibly long-term, changes in neurocognitive function are not nearly that simple, with neuroinflammation secondary to surgical-induced peripheral inflammation and availability of neuronal metabolic substrate appearing to play ever more important roles. This raises important questions as to what the most appropriate and effective strategies are to minimize risk. Such strategies will necessarily entail clearly identifying those most at risk and optimizing those factors which are modifiable (ex., minimizing pre-operative polypharmacy, use of non-opioid analgesics postoperatively). Whether EEG-guided changes in anesthetic administration will be an effective strategy is an ongoing question as is whether anesthetic adjuncts such as dexmedetomidine (or other agents) will be protective also remains to be answered. Finally, the question of whether surgery should be undertaken in the first place also needs close examination. While we have come far since Morton administered that first anesthetic in 1846, the words of the poet Robert Frost still resonate:

*The woods are lovely, dark and deep*,

*But I have promises to keep*,

*And miles to go before I sleep*,

*And miles to go before I sleep.*

## Author contributions

SS drafted the manuscript and prepared all figures. All authors contributed to the literature review and manuscript revision, and read and approved the submitted version.

## Conflict of interest

For full disclosure, PG serves on the scientific advisory board of Akelos, Inc., (New York, NY, United States), a biotechnology startup, for work unrelated to this work. The remaining authors declare that the research was conducted in the absence of any commercial or financial relationships that could be construed as a potential conflict of interest.

## Publisher’s note

All claims expressed in this article are solely those of the authors and do not necessarily represent those of their affiliated organizations, or those of the publisher, the editors and the reviewers. Any product that may be evaluated in this article, or claim that may be made by its manufacturer, is not guaranteed or endorsed by the publisher.
